# Cardiovascular risk reduction in African Americans: Current concepts and controversies

**DOI:** 10.21542/gcsp.2016.2

**Published:** 2016-03-31

**Authors:** Keith C. Ferdinand

**Affiliations:** Tulane Heart and Vascular Institute, Tulane University School of Medicine, 1430 Tulane Avenue, SL-8548, New Orleans, LA 70112, USA

## Introduction

Atherosclerotic cardiovascular disease (ASCVD) is the leading cause of death worldwide and in the United States (US). Health, life expectancy, and medical care have improved dramatically in the US over the last century. However, the distribution of benefits arising from the decreases in heart disease and stroke has not occurred equitably. The current mortality gap between blacks and whites in the US, although decreasing, has been persistent since 1960^1^. Black men and women are more likely to die of heart disease and stroke than white men and women. This population remains at higher risk for hypertension, type 2 diabetes, obesity (especially in females), myocardial infarction, stroke morbidity and mortality, chronic kidney disease (CKD), end-stage renal disease (ESRD), and cardiovascular mortality^2^.

## Race as a social concept and disparities in US life expectancy

Although this review specifically describes the extent and severity of ASCVD and associated risk factors in blacks, race is not a true biological or scientific category. While there may be individual aspects of biology and genotype that contribute to increased burden of heart disease and stroke in African Americans, chronic diseases are usually greatly impacted by environmental factors such as adverse diet, lifestyle, socioeconomic status, and exposures that lead to stress in disadvantaged areas. Patients, regardless of race or ethnicity, manifest disease within a cultural context. Hence, specific aspects of the patients’ disease burden are greatly impacted by family, health care providers, the community, and the health care system at large^3^.

**Figure 1.**
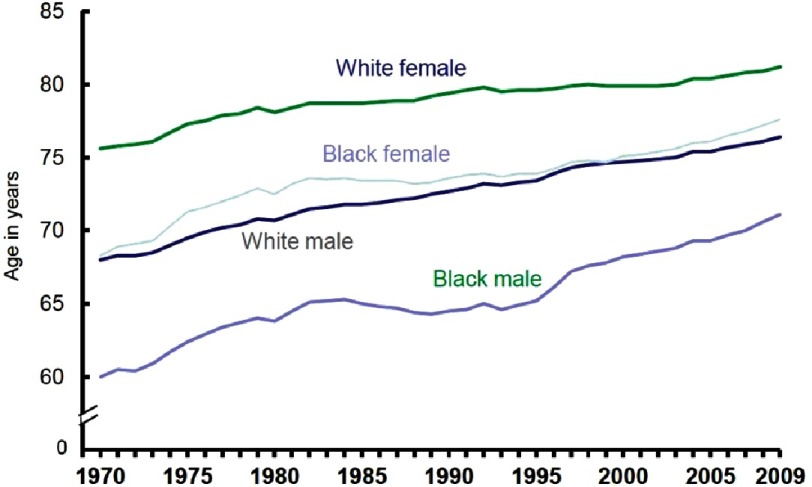
US life expectancy by race and gender^5^.

Most importantly, this excessive burden of heart disease and stroke in US blacks accounts for the largest portion of inequality in life expectancy between whites and blacks, despite existence of low-cost, highly effective preventive treatment (Figure 1).

**Figure 2.**
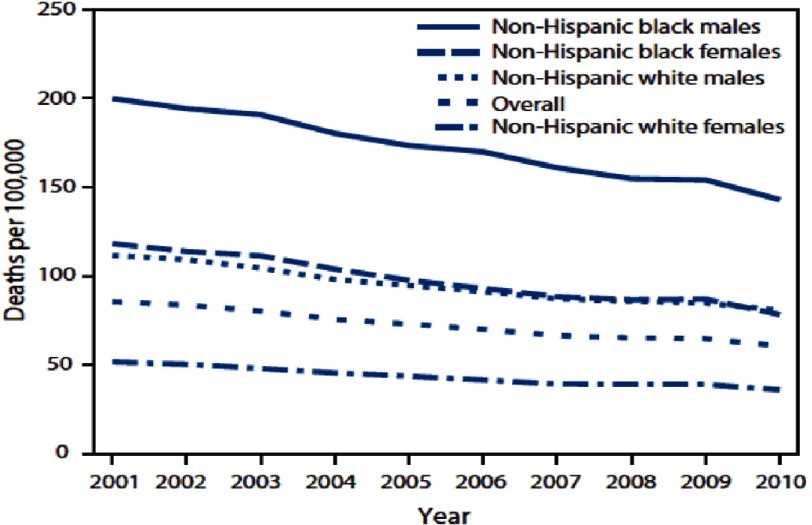
Avoidable death from heart disease, stroke, and CVD 2001–2010^6^.

In March of 1966, while speaking at the Medical Committee for Human Rights in Chicago, Illinois, Martin Luther King Jr. said, “Of all the forms of inequality, injustice in health care is the most inhumane.”^4^ In consideration of the above, cardiovascular disease (CVD) disparities by race and ethnicity are sizeable, likely multifactorial, and preventable (Figure 2).

The burden of ASCVD is primarily a result of excessive levels of cardiac risk factors, which are often preventable. Nevertheless, the social determinants of health are the circumstances in which people are born, grow up, live, work, and age, as well as the systems put in place to deal with illness. These circumstances are in turn shaped by a wider set of forces: economics, social policies, and politics. A recent American Heart Association (AHA) Scientific Statement confirmed the impact of these social determinants on disparate CVD burden^7^.

## Cardiometabolic risks in African Americans

**Figure 3.**
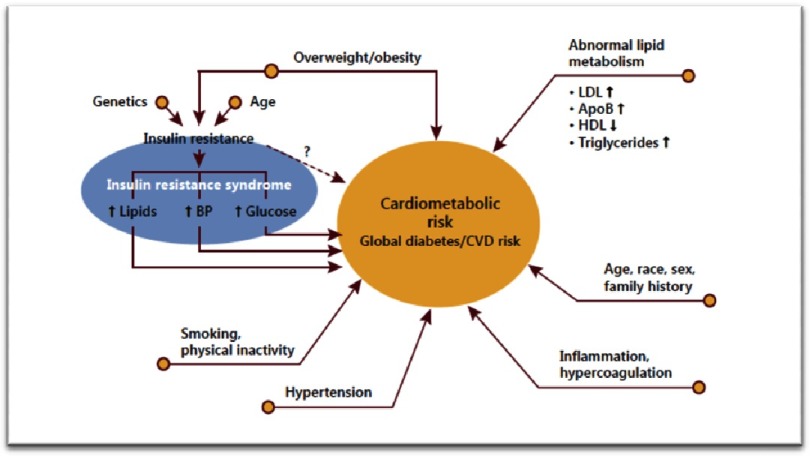
Contributions to CMR, global diabetes and CVD risk^8^.

The multiple conditions or behaviors that significantly contribute to CVD morbidity and mortality not only include traditional CVD risk factors, but also encompass a constellation of conditions termed cardiometabolic risk (CMR). The concept of CMR incorporates a wide range of characteristics such as obesity, abnormal lipid metabolism, insulin resistance, smoking, physical inactivity, hypertension, inflammation, hypercoagulation, and patient history (Figure 3)^8^.

**Figure 4.**
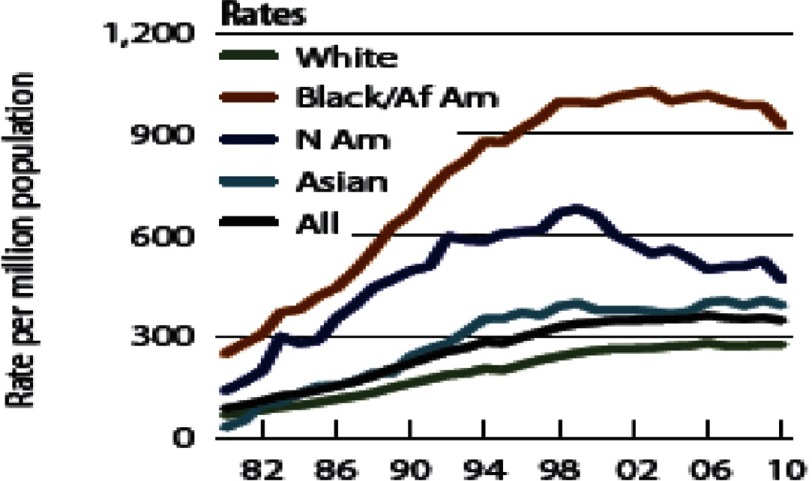
Rates of kidney disease by race/ethnicity. End-stage renal disease incidence has risen significantly among African-Americans^9^.

Hence, based on a variety of conditions as depicted below, African Americans show increased incidence of target organ damage associated with stroke, myocardial infarction mortality, left ventricular hypertrophy, heart failure, retinopathy, CKD, ESRD, and premature onset of morbidity and mortality (Figure 4)^9^.

A study by Heidenreich et al. demonstrates the projected percentage of heart failure according to race. The data show that by 2030, the projected risk of heart failure will increase to above 3.5% in US blacks, while the projected percentage is significantly less for US whites at 2.7%^10^. Perhaps one of the primary factors for the increase in ASCVD and CMR in US blacks is the increased rate of overweight and obesity status. African Americans have the highest rates of obesity in the US: 39% of black men and 57% of black women are considered obese. Additionally, 7% of black men and 17% of black women are considered morbidly obese (BMI >40). Among Mexican-Americans, another often disadvantaged ethnic minority, 38% of men and 43% of women are obese. Whites have the lowest obesity rates with 35% of white men and 34% of white women considered obese (Table 1)^11^.

**Table 1.** Prevalence of overweight and obesity in the US 2007–2012^11^.

Sociodemographic Characteristics of Person 25 Years or Older From the NHANES, 2007-2012^a^								
			Percentage					
	Study Population, No.					Obesity Class		
Characteristic	Sample	Weighted	Underweight	Normal weight	Overweight	1	2	3
**Men**								
Race/ethnicity								
Mexican American	1845	12316214	0.35	18.75	43.17	24.83	8.21	4.70
Non-Hispanic black	1577	9245105	1.73	25.67	33.44	21.80	9.90	7.46
Non-Hispanic white	3427	63145888	0.62	23.35	40.74	23.36	7.80	4.13
Other	629	6187710	1.36	42.33	35.47	15.58	2.09	3.17
Age,y								
25-54	4143	59105817	0.69	25.05	39.38	22.51	7.78	4.59
≥55	3335	31789101	0.84	22.78	41.05	23.54	7.50	4.29
**Women**								
Race/ethnicity								
Mexican American	2024	11983246	0.68	22.43	33.58	24.16	12.34	6.81
Non-Hispanic black	1653	11484735	1.66	15.79	25.77	26.03	13.45	17.30
Non-Hispanic white	3417	67131553	2.28	33.77	30.02	17.58	9.37	6.98
Other	636	6556840	3.23	50.02	26.84	10.80	4.76	4.35
Age,y								
25-54	4291	59578408	2.29	33.45	28.58	17.64	9.82	8.22
≥55	3439	37577965	1.73	28.01	31.58	20.98	10.05	7.65

**Notes.**

TITLE NHANES National Health and Nutrition Examination Survey.

a Patients are divided by National Heart, Lung, and Blood Institute recommaended weight group and sex. Weight groups were defined by body mass index (calculated as weight in kilograms divided by height in meters squared): underweight (<18.5), normal weight (18.5-24.9), overweight (25.0-29.9), obesity class 1 (30.0-34.9), obesity class 2 (35.0-39.9), and obesity class 3 (≥40).

**Figure 5.**
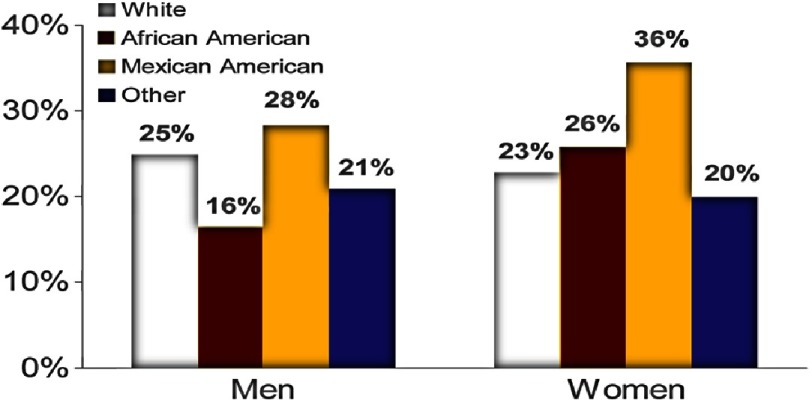
Prevalence of the National Cholesterol Education Program’s metabolic syndrome NHANES III by sex and race/ethnicity^13^.

Surprisingly, according to the National Health and Nutritional Examination Survey (NHANES), blacks have lower triglycerides and higher high density lipoprotein-cholesterol (HDL-C). Thus, prevalence of metabolic syndrome defined by arbitrary cut-points is actually determined as lower in blacks than whites (Figure 5)^12^.

These racial differences in metabolic syndrome prevalence, in view of lower triglyceride levels and relatively higher HDL-C, are more prominent in men than in women. Considering higher rates of stroke and myocardial infarction in blacks versus whites, the favorable lipid profile of low triglycerides and high HDL-C in blacks is both surprising and paradoxical. Therefore, the widespread use of triglyceride levels to predict insulin resistance, CVD, and type 2 diabetes needs reevaluation. Yu and colleagues have coined the term triglyceride paradox in people of African descent^12^.

Safford et. al. conducted a study investigating the race-sex differences in the management of hyperlipidemia. Since coronary heart disease (CHD) risk influences physician prescribing, Adult Treatment Panel III CHD risk categories were constructed using baseline data from Reasons for Geographic and Racial Differences in Stroke study participants (recruited 2003–2007). Prevalence, awareness, treatment, and control of hyperlipidemia were examined for race-sex groups across CHD risk categories. Multivariable models conducted in 2013 estimated prevalence ratios adjusted for predisposing enabling and need factors influencing health services utilization. The study described several potentially actionable findings that likely contribute to racial disparities in acute CHD. Black males were less likely to be aware of hyperlipidemia compared with other race-sex groups, supporting the need for interventions to improve their awareness. This study also found that among those at highest CHD risk, white males were significantly more likely to be treated and controlled, and blacks in general, but especially black women, were less likely to be treated or controlled^14^.

To date, the Antihypertensive and Lipid-Lowering treatment to prevent Heart Attack Trial (ALLHAT) lipid arm with pravastatin has the largest subpopulation of black patients, with non-Hispanic black patients accounting for one-third of the trial population; however, the clinical implications of the apparently favorable subgroup results in African American patients remain unclear^15^. Clinical-event trials comparing different statins have been predominantly white or European (approximately 90%) and the subgroup data are often inadequate or unavailable. Therefore, while the risk of CHD is clearly greater in African American men and women, not enough data exist to aid physicians in treating their African American patients to lower this risk (Table 2)^15–22^.

**Table 2.** Percentage of black patients in statin clinical event trials^15–22^.

Trial	Statin	Total Patients, n	African Americans, n or %
4S	Simvastatin	4444	N/A
WOSCOPS	Pravastatin	6595	N/A
CARE	Pravastatin	4159	Others, 7-8%
LIPIED	Pravastatin	9014	N/A
AFCAPS/TEXCAPS	Lovastatin	6605	206
HPS	Simvastatin	20536	N/A
ALLHAT	pravastatin	10355	3491
ASCOT	Atorvastatin	10305	Others, 5-5.5%

It is known that baseline creatine kinase (CK) levels are higher in African Americans than in whites and that they are higher in men than in women^23^. The Justification for the Use of Statins in prevention: An Intervention Trial Evaluating Rosuvastatin (JUPITER) found 12,683 whites and 5,117 non-whites had low density lipoprotein-cholesterol (LDL-C) less than 130 mg/dL and high-sensitivity C-reactive protein (hs-CRP) greater than or equal to 2.0 mg/L. Rosuvastatin significantly decreased the rate of first MI, stroke, arterial revascularization, hospitalization for unstable angina, and cardiovascular death among whites and nonwhites^24^. Therefore, JUPITER is a welcomed addition to clinical evidence on statin therapy in blacks.

Based on limited statistical power, lipoprotein(a) (Lp(a)) was previously not considered a risk factor for CVD in blacks. However, Lp(a) is significantly higher among blacks versus whites, and in both, increased Lp(a) correlates positively with LDL-C and negatively with triglycerides. Increasing quintiles of Lp(a) was equally as predictive of future CVD in blacks as in whites^25^.

The Coronary Artery Risk Development In Young Adults (CARDIA) study found that for blacks, LDL-C levels in are adults significantly lower (*P* < 0.001) with three genetic variations—L253F, C679X, and Y142X—(81.5 mg/dL) and A443T (95.5 mg/dL) compared to non-carriers (109.6 mg/dL). The three genetic variants and the A443T variant in black men associated with lower carotid intima media thickness and lower prevalence of coronary calcification measured at ages 38 to 50^26^.

## The Jackson Heart Study: A paradigm of cardiovascular risk assessment in African Americans

The Jackson Heart Study (JHS), a National Heart, Lung, and Blood Institute (NHLBI) sponsored epidemiologic study, followed 5,301 individuals since 2000. This landmark study seeks to explore the reasons for racial disparities in ASCVD and cardiac risk and to uncover new approaches to reduce them^27^. By identifying factors that influence the development and worsening of CVD, JHS would potentially be best able to prevent this high burden of ASCVD in US blacks in the future.

Distressingly, although 88% of JHS participants were ideal in risk factor status for smoking, fewer than half met the ideal criteria for fasting plasma glucose (45.3%) or total cholesterol (45.2%). Moreover, fewer than a quarter of the JHS cohort met the ideal criteria for physical activity (19.3%), blood pressure (17.8%), and BMI (13.7%), and only 0.9% met the ideal dietary intake^27^.

The very low prevalence of ideal cardiovascular health among participants of the JHS, and particularly the extremely low prevalence of ideal BMI, physical activity, and diet, underscores the need for reassessment of tools and strategies available to achieve the AHA 2020 goal among African Americans. In this manner, data from JHS also provide specific targets for clinical and public health interventions (i.e., diet, physical activity, and adiposity) as well as help to explain the burden of CVD in stroke-belt regions across the US. Identification of those harboring subclinical atherosclerosis will supplement current risk stratification strategies.

Perhaps due to the profound effects of hypertension in African Americans, despite having an observed lower frequency of elevated calcium artery calcification (CAC) scores, the survival rates remain disproportionally decreased when compared to other groups. The prevalence of CAC is 66% for non-Hispanic whites, 58% for African Americans, 55% for Hispanics and 55% for Asians (p<0.0001). As compared with non-Hispanic whites, all ethnic groups had lower odds of having an increasing burden of CAC, but blacks, nevertheless, had higher mortality rates^28^.

## Unique aspects of antihypertensive pharmacotherapy in blacks: What is the evidence?

Hypertension prevalence in African Americans is among the highest in the world^29–31^. Compared to US whites, blacks develop hypertension at an earlier age, and average blood pressure is much higher. Potential physiologic and hemodynamic determinants of this hypertension could be obesity, higher salt sensitivity, low levels of plasma renin, vascular function (sympathetic over-activity), attenuated nocturnal fall in blood pressure, greater comorbidity (especially with diabetes), inactivity, and family history^38–40^.

Materson et. al. conducted a study in 1993 to uncover the effects of age and race on the effectiveness of single-drug antihypertensive medications in US veterans. The study included six antihypertensive medications and a placebo, one of which was randomly given to each of the 1292 men. Of these men, 48% were black and their ages ranged from 59–69. Results of this study found that age and race are indeed influential factors in the effectiveness of certain antihypertensive medications. Black men, both young and old, responded best to treatment with diltiazem. However, younger whites responded best to treatment with captopril, and older whites with atenolol. Blacks did not respond as well to captopril. Therefore, the study demonstrated that age and race should be considered when making treatment decisions^32^.

These findings were supported by another study in 2004, the Losartan Intervention For Endpoint reduction in hypertension (LIFE) study, which compared treatment with losartan and atenolol in black and non-black patients with hypertension and left ventricular hypertrophy (LVH). The study found that blacks had better outcomes after treatment with atenolol regimens, while the opposite was true for non-blacks in whom losartan was superior^33^.

**Figure 6.**
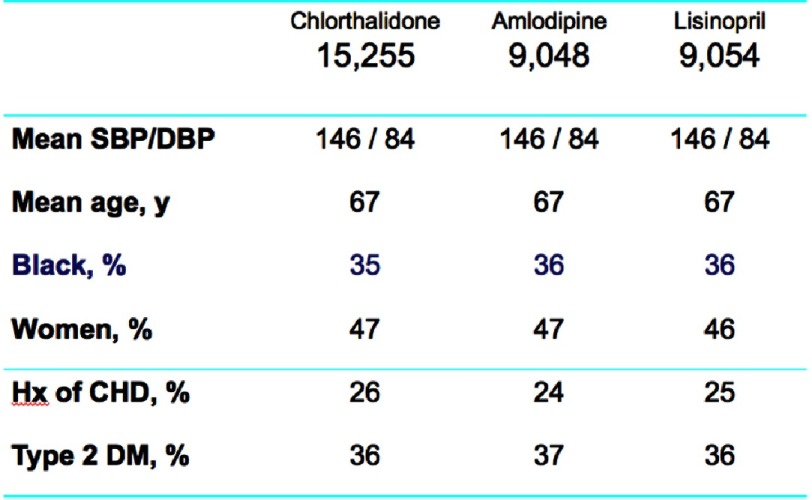
Baseline characteristics for ALLHAT participants^34^.

The NHLBI sought out to further investigate racial differences in the pharmacologic treatment of hypertension. The ALLHAT study was initiated in over 42,000 patients at high risk for elevated blood pressure. The proportion of blacks was approximately 35%, which enabled the investigators to compare black versus non-black participants in response to medications and CVD endpoints (Figure 6)^34^.

**Figure 7.**
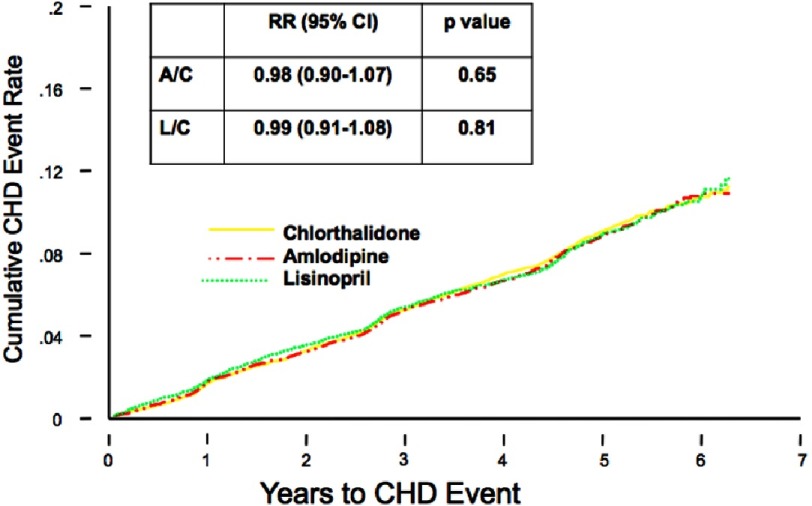
Primary outcome (fatal CHD or nonfatal MI) by treatment group^45^.

**Figure 8.**
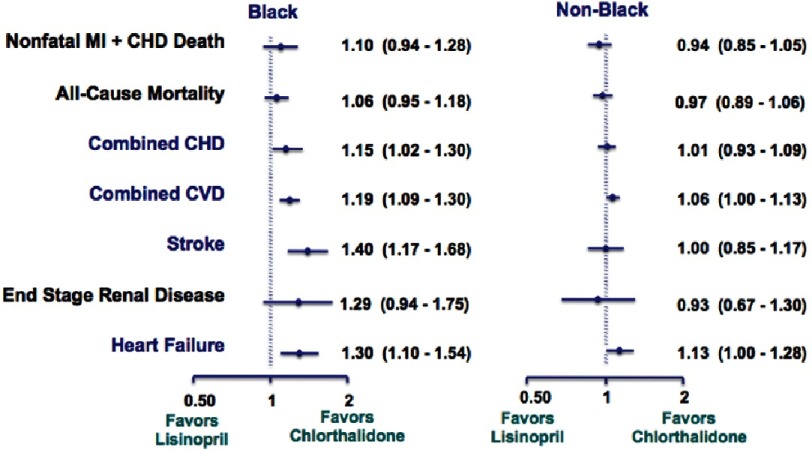
Black versus non-black lisinopril vs. chlorthalidone^35^.

**Figure 9.**
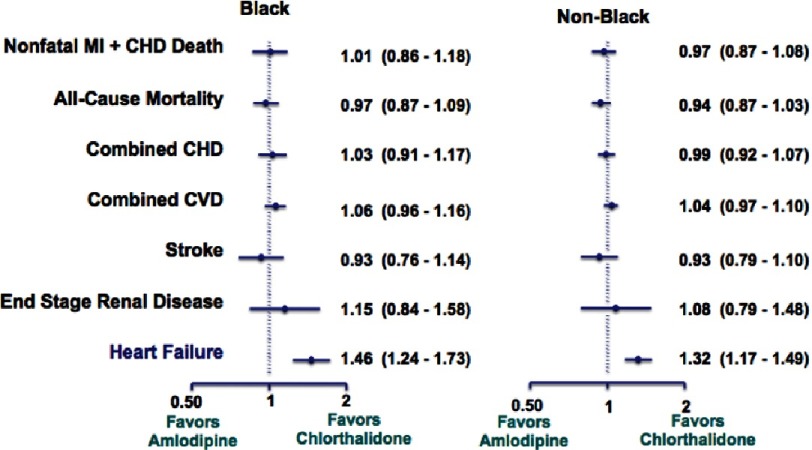
Black versus non-black amlodipine vs. chlorthalidone^35^.

**Figure 10.**
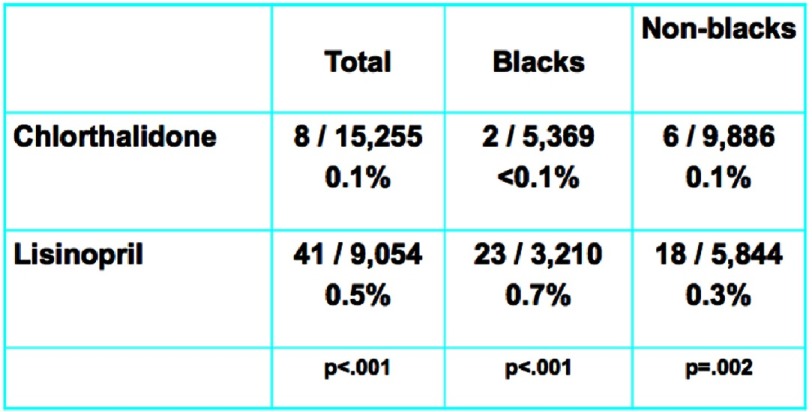
Angioedema differences in chlorthalidone vs. lisinopril^35^.

Chlorthalidone, amlodipine, and lisinopril were shown to have no significant difference in the primary endpoint effect on CHD event rate, but demonstrated differences were noted among certain diseases (Figure 7). In the lisinopril–chlorthalidone comparison, there were no differences in either sub-group for major CHD or total mortality. However, there were some differences by race, primarily for stroke, for which lisinopril-based treatment was significantly less effective in blacks by 40%. Heart failure was also prevented more effectively by chlorthalidone-based treatment than lisinopril, and the magnitude of this effect was not significantly different by race. Primarily as a consequence of these outcomes, combined CVD was lower in the chlorthalidone group for both races, although the effect was more pronounced in the black sub-group. Interestingly, the risk of ESRD was not reduced by lisinopril compared with chlorthalidone in either sub-group, although the confidence limits were somewhat wide (Figures 8 and 9). As well, while all patients experienced less angioedema with treatment with chlorthalidone compared to lisinopril, blacks had significantly higher rates of angioedema due to lisinopril compared to whites (Figure 10)^35^.

**Figure 11.**
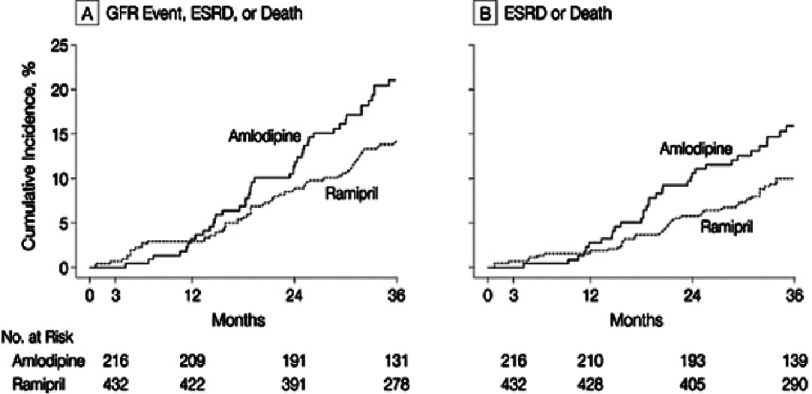
AASK outcomes^36^.

On the other hand, in blacks, the benefits of angiotensin converting enzyme inhibitors (ACEIs) have been shown in CKD. A study on the effects of ramipril and amlodipine on renal outcomes in hypertensive nephrosclerosis was conducted by the African American Study of Kidney Disease and Hypertension (AASK) study group. The study found that those treated with ramipril were at lower risk both initially and in the long-term for a glomerular filtration rate (GFR) event, ESRD, or death when compared to those treated with amlodipine^36^ (Figure 11).

Initial medications for the management of hypertension in blacks should include a thiazide-type diuretic and/or a calcium channel blocker (CCB). Most patients will require a two or three-drug combination to control their hypertension. If a diuretic is contraindicated or not tolerated, a CCB plus an ACEI or angiotensin receptor blocker (ARB) is required. However, ACEIs (and likely other RAS blockers) are less effective than other classes in lowering BP and preventing cardiovascular events in African Americans. For this reason, ACEIs are not the preferred initial agents unless combined with a thiazide-type diuretic or CCB. Chlorthalidone 12.5–25 mg/d is the thiazide-type diuretic of choice, especially is high risk blacks, since it is more potent and longer-acting than hydrochlorothiazide. Based on ALLHAT results and its long action and high tolerability, amlodipine is the CCB of choice. A loop diuretic (e.g., furosemide) may be needed with advanced CKD (eGFR <30). Regardless of needed pharmacotherapy, lifestyle modifications such as a low sodium diet, DASH eating plan, and adequate physical activity are the bedrock of appropriate hypertension therapy. Beta-blockers should be included in the regimen if there is compelling indication for a beta-blocker, such as recent myocardial infarction or heart failure with reduced ejection fraction^37^.

## Treatment of resistant hypertension

Resistant hypertension is blood pressure above goal despite adhering to full doses of an appropriate three-drug antihypertensive regimen including a diuretic. Withdrawal or down titration interfering substances use adequate long-acting thiazide, preferably chlorthalidone, and combine different mechanisms of action. The recommended treatment of resistant hypertension includes a triple regimen of ACEIs (or ARB), CCB, and thiazide diuretic. Additional effects of BP reduction have also been shown with spironolactone in resistant hypertension^38^. In view of approximately half of adults with hypertension in the US are uncontrolled, perhaps, 12 % to 15 % are considered resistant. Moreover, compared with men, women have more prevalent resistant hypertension with greater older age status and obesity, two of the strongest predictors. Resistant hypertension is more common in African Americans and more frequent with conditions such as heart disease, heart failure, diabetes, stroke, and CKD. All patients require lifestyle modifications and effective treatment mandates combination therapy, including appropriate diuretics, renin-angiotensin system blockers, CCB, and underutilized aldosterone antagonists. In the future, emerging, innovative interventions may include renal artery denervation and carotid stimulation treatment, which are potentially effective approaches to BP control^39^.

## Eliminating disparities

Efforts for the elimination of disparities can come in many forms, such as raising public and provider awareness of racial and ethnic disparities in health care, expanding health insurance coverage, improving capacity and number of providers in underserved communities, and increasing the knowledge based on causes and interventions to reduce disparities^40^. Moreover, the Centers for Disease Control (CDC) and Centers for Medicare and Medicaid Services (CMS) came together to create and lead the Million Hearts^®^ initiative. This initiative, which was launched in 2012, aims to prevent one million heart attacks and strokes by 2017. The Million Hearts^®^ approaches the ABCs of eliminating disparities that include appropriate aspirin therapy for those who need it, BP control, cholesterol management, and smoking cessation^41^. The National Forum for Heart Disease and Stroke Prevention, a coalition of over 80 organizations, aims to eliminate disparities and addresses the most significant population-level interventions to prevent heart disease, including reduction of sodium intake and tobacco use. In the US population, consumption of sodium far surpasses safe levels and contributes to hypertension, a great deal of which is uncontrolled. By lowering the amount of sodium in processed foods and products used for food preparation in the service industry, the consumption of sodium by the population will decrease. Overall, the US population consumes a diet consisting of excessive sodium and sugar intake and lacking in fish, fresh fruits, and vegetables. To better prevent the incidence of heart disease and stroke, the prevailing typical American diet, high in saturated fat, sugar and sodium, needs to be reconsidered^42^.

There is a need to develop, implement, and evaluate interventions to prevent CVD and risk factors. The department of Health and Human Services (HHS) will implement interventions ranging from quality of care improvements to potential reimbursement incentives for policy and health system changes, working both with minority providers and providers serving minority populations. These efforts will expand upon outreach toward minorities and aid in the adoption of certified electronic health records (EHR)^43^.

At the individual level, influences include biological effectiveness of medications, adherence to medications/lifestyle, mental health and substance abuse, reactions to discriminations, health literacy, English proficiency, employment status, and health insurance coverage. At the provider/clinical team level, influences include knowledge, communication skills, awareness of disparities, cultural competency, and trustworthiness^44^. Almost half (48%) of patients with hypertension or diabetes have inadequate health literacy, and therefore lack knowledge about their disease, important lifestyle modifications, and essential self-management skills^45^. Furthermore, evidence suggests that the issue of disparities in health outcomes of CVD, including acute coronary syndromes and chronic conditions, is not simply a result of the bias of clinicians, but rather additionally a “systems” problem that can be abolished with adequate performance improvement initiatives.
